# Sex Differences in the Association Between the Korean Healthy Eating Index and Liver Enzymes Among Korean Adults

**DOI:** 10.3390/nu17142372

**Published:** 2025-07-20

**Authors:** Seong-Uk Baek, Jin-Ha Yoon

**Affiliations:** 1 Graduate School, Yonsei University College of Medicine, Seoul 03722, Republic of Korea; 2 Department of Preventive Medicine, Yonsei University College of Medicine, Seoul 03722, Republic of Korea

**Keywords:** healthy behavior, healthy diet, healthy eating, healthy eating index

## Abstract

Background/Objectives: Dietary quality plays a crucial role in maintaining liver function. In this study, we examined sex differences in the association between dietary quality and elevated liver enzyme levels in Korean adults. Methods: This study included a nationwide sample of 15,997 males and 22,300 females in South Korea. Dietary assessment was performed using the Korean Healthy Eating Index (KHEI), an evidence-based dietary quality index that quantitatively reflects adherence to Korean dietary guidelines (range 0–100). Serum levels of aspartate aminotransferase (AST) and alanine aminotransferase (ALT) were measured, with individuals classified as having elevated AST or ALT levels when values were ≥40 IU/L or ≥35 IU/L, respectively. Logistic regression analysis was performed to examine the association between the KHEI and elevated AST or ALT levels stratified by sex. Odds ratios (ORs) and 95% confidence intervals (CIs) were determined. Results: Among the males, a 10-point increase in the KHEI score was inversely associated with the likelihood of having elevated AST (OR: 0.90; 95% CI: 0.85–0.96) and ALT (OR: 0.96; 95% CI: 0.92–1.00) levels, respectively. Among the females, there was no clear association between the KHEI and elevated AST (OR: 0.98; 95% CI: 0.91–1.05) or ALT (OR: 1.00; 95% CI: 0.95–1.05) levels. Conclusion: Further research is warranted to elucidate the underlying mechanisms of the observed sex-specific associations and guide the development of targeted dietary interventions for liver health in males and females.

## 1. Introduction

Promoting a healthy diet is crucial for preventing cardiovascular and metabolic diseases. According to a 2017 Global Burden of Disease study, approximately 11 million deaths worldwide can be attributed to dietary risk factors, including excessive sodium intake and insufficient consumption of whole grains and fruits [1]. In addition, low intake of vegetables, omega-3 fatty acids, milk, nuts or seeds, and polyunsaturated fats, along with high intake of trans fats, sugar-sweetened beverages, and processed or red meat, also contributes to increased disease burden [1]. In South Korea, dietary risk factors such as low consumption of whole grains and fruits and high sodium intake significantly contributed to mortality associated with cardio-metabolic disorders [2].

Dietary quality plays an important role in the maintenance of liver function [3]. Recently, high dietary quality has been shown to be associated with a reduced risk of metabolic dysfunction-associated steatotic liver disease [4,5,6,7]. Furthermore, high dietary quality has been prospectively associated with a reduced risk of mortality related to chronic liver diseases [8,9]. Poor diet quality, as determined by the Healthy Eating Index or Mediterranean-style diet score, can cause hepatic steatosis and systemic inflammation, which may contribute to elevated liver enzymes [3,10]. Western-style dietary patterns are reportedly linked to increased alanine aminotransferase (ALT) levels in individuals with dyslipidemia or impaired fasting glucose [11]. Genetic predisposition also plays an important role in the relationship between diet and liver disease. For instance, certain genetic factors influence the severity and natural history of steatotic liver disease and hepatitis [12]. Recent studies have indicated that the impact of diet on hepatic steatosis and inflammation is markedly pronounced among those with genetic risks [13,14].

Notable sex-based dimorphisms have been identified in the prevalence and pathophysiology of liver disease. Studies have well-documented sex-based differences in fat distribution, with women tending to accumulate more subcutaneous fat compared to men, who are more likely to accumulate visceral fat [15,16]. Liver steatosis tends to be more prevalent in males than in females, possibly due to the role of sex hormones such as estrogen and androgens, which influence lipid metabolism and inflammation in liver tissue [17,18]. Although a link between dietary quality and abnormal liver function has been previously reported, sex differences in this association remain poorly investigated. While a Western dietary pattern was found to be associated with elevated ALT levels among females, a Mediterranean dietary pattern was inversely associated with gamma-glutamyl transferase among males [11]. Identifying sex differences in the association between dietary quality and liver enzyme levels may offer a more nuanced understanding of the relationship between diet and liver health, with meaningful implications for clinical practice and public health promotion.

Therefore, in the current study, we aimed to examine sex differences in the association between dietary quality as measured using the Korean Healthy Eating Index (KHEI) and liver enzyme levels using a nationally representative sample from the Korea National Health and Nutrition Examination Survey (KNHANES).

## 2. Materials and Methods

### 2.1. Study Population

This study utilized data from the 2013–2021 survey years of the KNHANES, a nationwide health examination survey targeting the general population in Korea. The KNHANES is conducted annually by the Korea Disease Control and Prevention Agency (KDCA). A nationally representative sample was selected using systematic sampling, in which households were drawn from 516 administrative districts across the country [19]. The selected households underwent face-to-face interviews and health examinations in mobile examination vehicles. The response rates for the KNHANES were 78.3% for 2013–2015, 76.6% for 2016–2018, and 74.0% for 2019–2021 [20]. Raw data from the KNHANES are available at https://knhanes.kdca.go.kr/ (access date: 1 May 2025). The 2013–2021 KNHANES was conducted with the ethical approval of the Institutional Review Board of the KDCA (2013-07CON-03-4C; 2013-12EXP-03-5C; 2018-01-03-P-A; 2018-01-03-C-A; 2018-01-03-2C-A; 2018-01-03-5C-A).

**Figure 1:**
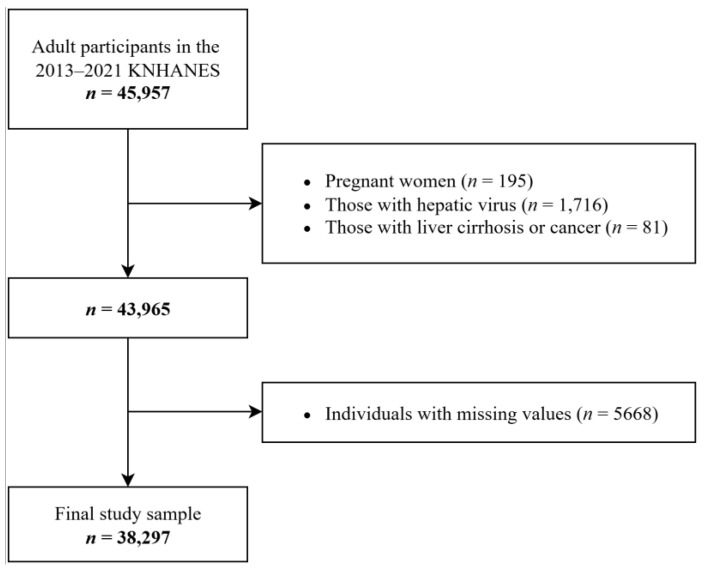
Diagram illustrating the sample selection procedure.

Figure 1 presents a diagram of the study sample selection process. In total, 45,957 individuals aged > 18 years were included in the 2013–2021 survey years of the KNHANES. Pregnant women (*n* = 195), individuals with hepatic viruses B or C (*n* = 1716), and individuals diagnosed with liver cirrhosis or cancer (*n* = 81) were excluded from the study. Finally, 38,297 adults were included in the final sample after excluding those with missing data (*n* = 5668).

### 2.2. KHEI

The KHEI is used to assess the dietary quality of the Korean population. It was developed by the KDCA based on the Healthy Eating Index of the United States Department of Agriculture [21], adapting it to Korean dietary habits and culture [22]. The KHEI comprises fourteen items: eight assess the adequacy of food intake, such as vegetables, whole grains, fruits, meat, fish, eggs, beans, or milk; three evaluate the moderation of components such as saturated fatty acids, sodium, and sweets; and three measure the balance of energy intake from carbohydrates, fats, and total energy. Specifically, the breakfast score was determined based on the number of days breakfast was consumed per week, while the scores for mixed grains, total fruits, fresh fruits, total vegetables, meat, fish, eggs, and beans were calculated based on the number of servings consumed per day for each food item. The detailed scoring system has been elaborated on in previous literature [22]. The composite KHEI ranged from 0 to 100, with higher scores indicating better dietary quality. Based on quintiles of the KHEI in the overall sample, individuals were categorized into five groups: lowest dietary quality (<51.5), low dietary quality (51.5–60.1), average (60.1–67.1), high (67.1–74.8), and highest dietary quality (>74.8). Dietary assessment was conducted using the 24-hour recall method. For more accurate measurements, professional nutritionists assisted with the dietary recall. Nutrient intake was determined using the standard food composition table [23].

### 2.3. Elevated Liver Enzymes

The study participants underwent blood sampling via venipuncture after fasting for at least 8 h. To determine serum levels of aspartate aminotransferase (AST) and ALT, the ultraviolet method without pyridoxal-5-phospate was applied using a Labospect 008AS analyzer (Hitachi, Tokyo, Japan). According to the cutoff values for the Korean population [24,25], elevated AST and ALT levels were defined as ≥40 IU/L and ≥35 IU/L, respectively, regardless of age or sex.

### 2.4. Covariates

The following sociodemographic features were adjusted in the models: Age (in years) was adjusted as a continuous scale. Income levels were grouped into four categories based on the quartile values of total household income: lowest, low, high, and highest. Educational attainment was grouped as middle school or below, high school, and college or above. Marital status was categorized as married, unmarried, or other (separated, widowed, or divorced). Employment status was grouped into worker and unemployed. Current smoking status was classified as no or yes. Physical activity was categorized as “yes” if individuals engaged in ≥150 min per week of moderate to vigorous physical activity and “no” otherwise. Excessive alcohol consumption was defined as an alcohol intake of ≥20 g/day for males and ≥10 g/day for females. This threshold was selected because it has been widely adopted as the standard level of alcohol intake shown to significantly affect liver function in studies assessing alcohol-related liver outcomes, particularly in relation to steatotic liver disease [26,27]. This cutoff also corresponds to the level proposed by the Asian Pacific Association for the Study of the Liver [26]. The body mass index was included as a continuous variable. Hypertension and diabetes were each classified as “yes” or “no.” The presence of hypertension was determined based on current use of antihypertensive medications, and the presence of diabetes was determined based on current use of antidiabetic drugs or insulin.

### 2.5. Statistical Analysis

Descriptive analyses were used to examine the sociodemographic features of the study participants, as well as the distribution of the total and subcomponent KHEI scores among the study participants. Logistic regression analysis was performed to determine the association between KHEI and elevated AST and ALT levels. Specifically, we investigated how a 10-point increase in the KHEI score was associated with changes in the likelihood of elevated AST or ALT levels by calculating the odds ratios (ORs) and 95% confidence intervals (CIs). Sex-stratified analyses were conducted to explore whether the associations between KHEI and elevated AST or ALT levels differed by sex. In addition, an interaction term between KHEI and sex was included in the regression model to assess the statistical significance of sex differences in these associations. Additionally, in the logistic regression models, the main independent variable, KHEI, was scaled in units of 10 to examine whether a 10-point increase was associated with a change in the odds of having elevated AST or ALT levels. To verify the robustness of our findings, multiple imputations were used to handle the missing values in the datasets. Twenty datasets with complete information were generated through multiple imputations using the chained equation (MICE) method, and the estimates were combined and presented [28].

## 3. Results

The sociodemographic characteristics of the participants are shown in Table 1. A total of 15,977 males and 22,300 females were included in this study. The mean (standard deviation [SD]) age of the male and female participants was 51.5 (17.1) and 51.4 (16.4), respectively. The mean (SD) KHEI scores were 61.6 (13.0) for the males and 64.1 (13.5) for the females. Overall, 7.1% (*n* = 1131) of the males and 3.4% (*n* = 751) of the females had elevated AST levels, whereas 19.5% (*n* = 3126) of the males and 6.7% (*n* = 1493) of the females had elevated ALT levels. The frequencies of elevation of both AST and ALT levels were significantly higher in the male group than in the female group. Women had higher scores than men in overall KHEI, as well as in the total adequacy, moderation, and balance components.

The distribution of the KHEI subcomponents is presented in Table 2. The mean (SD) KHEI values across the dietary quality categories (lowest, low, average, high, and highest) were 43.6 (6.2), 55.9 (2.5), 63.6 (2.0), 70.6 (2.2), and 80.5 (4.6) for men and 43.6 (6.4), 56.0 (2.4), 63.5 (2.0), 70.8 (2.2), and 81.3 (5.0) for women. The mean total adequacy, moderation, and balance scores were higher in the high dietary quality category. Among men, the highest levels of AST, ALT, and BMI were observed in the lowest KHEI group, whereas the lowest levels were observed in the highest group. In contrast, among women, the highest levels of AST and ALT were observed in the highest KHEI group, while the lowest levels were observed in the lowest group.

The sex difference in the KHEI subcomponents is presented in Table S1. Women had higher total KHEI, adequacy, and moderation scores than men. Specifically, they consumed more fruits and dairy and had better sodium moderation. In contrast, men had higher intake scores for vegetables and protein-rich foods such as meat, fish, eggs, and beans.

BMI demonstrated a statistically significant positive correlation with both AST (r = 0.34, *p* < 0.001) and ALT (r = 0.16, *p* < 0.001) levels, as determined by Pearson’s correlation coefficient. Furthermore, BMI had a negative correlation with KHEI score (r = −0.02, *p* < 0.001).

Among the males, higher dietary quality was associated with lower odds of elevated AST levels (Table 3). Compared to those with the lowest dietary quality, the adjusted ORs (95% CIs) for elevated AST were 0.98 (0.80–1.20) in the low, 0.76 (0.60–0.96) in the average, 0.81 (0.64–1.01) in the high, and 0.67 (0.52–0.88) in the highest dietary quality groups. Additionally, each 10-point increase in the KHEI score was associated with a 10% reduction in the odds of elevated AST levels (OR: 0.90; 95% CI: 0.85–0.96). In contrast, no clear association was observed between KHEI and elevated AST levels in the females. A similar pattern was observed for ALT levels among the males. Compared to those with the lowest dietary quality, the adjusted ORs (95% CIs) for elevated ALT were 0.98 (0.85–1.13), 0.91 (0.79–1.06), 0.97 (0.83–1.12), and 0.80 (0.68–0.96) across increasing dietary quality categories. A 10-point increase in the KHEI score was also associated with a modest reduction in the odds of elevated ALT levels (OR: 0.96; 95% CI: 0.92–1.00). However, this association was not evident in the females.

Table S2 shows that the interactions between the KHEI and sex in relation to elevated AST or ALT levels were significant at *p* = 0.001 for AST and *p* < 0.001 for ALT.

**Figure 2:**
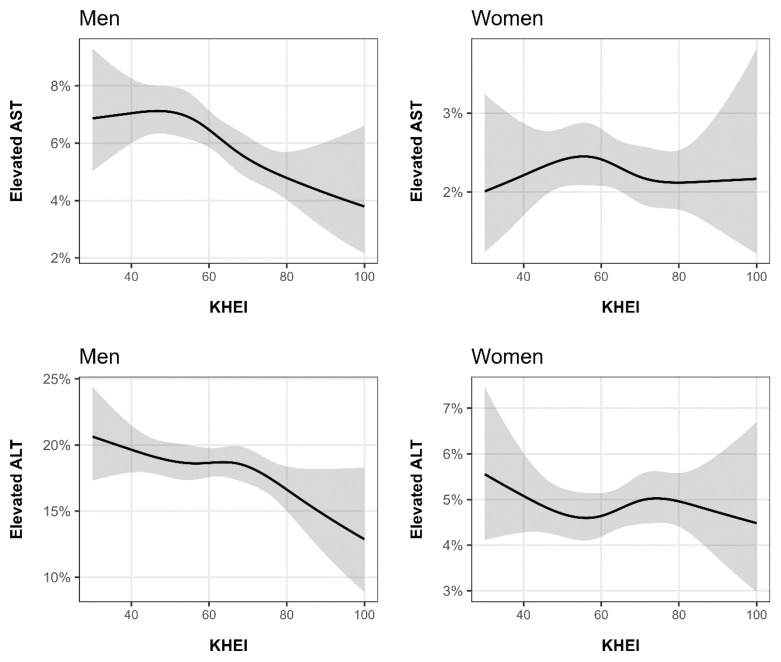
Nonlinear association between the Korean Healthy Eating Index and elevated serum aspartate aminotransferase (AST) and alanine aminotransferase (ALT) levels in men and women. The *y*-axis represents the predicted probability of elevated AST or ALT levels, while the *x*-axis indicates the KHEI score.

Figure 2 illustrates the nonlinear association between the KHEI and elevated AST and ALT levels. Among the males, a decreasing trend in the predicted probability of elevated AST levels was observed, beginning at a KHEI score of approximately 60, with a similar decreasing trend for elevated ALT levels noted from a score of approximately 70. In contrast, there was no clear increasing or decreasing trend in the association between KHEI scores and AST or ALT levels in the females.

Sensitivity analyses using the MICE method yielded results similar to those observed in the main analyses (Table S3).

## 4. Discussion

The primary finding of this study was the sex-based differences in the association between KHEI and elevated AST and ALT levels, wherein higher dietary quality was associated with a lower likelihood of elevated AST or ALT levels, especially among males. However, no clear association was identified in females.

Our findings are consistent with those of previous studies showing an inverse association between dietary quality and elevated liver enzyme levels [29,30]. Dietary quality is inversely associated with the risk of steatotic liver disease [4,31,32,33]. Regarding individual dietary components, a sufficient intake of vegetables and fruits was associated with lower levels of both AST and ALT [34], whereas consumption of foods high in fat or sugar was linked to elevated liver enzyme levels [35,36]. In addition, an intervention study revealed that consuming a well-balanced lunch could reduce ALT levels among Japanese workers [37].

Complex mechanisms may explain the negative association between dietary quality and the low prevalence of elevated liver enzyme levels. It is well established that a healthy diet plays a crucial role in improving an individual’s metabolic profile [38,39]. A high KHEI score has been associated with regular breakfast consumption; diverse and abundant intake of fruits and vegetables; adequate consumption of protein-rich foods such as meat and eggs; and moderate consumption of carbohydrates, fats, sugars, and sodium. These dietary habits may help prevent fat accumulation and inflammatory changes in the liver, thereby reducing hepatic cell damage and suppressing elevated AST and ALT levels [40,41,42].

As stated, the main finding of this study was the presence of sex-related differences in the association between dietary quality and liver enzyme levels. Although the exact mechanisms have not been fully elucidated, differences in genetic predispositions and sex hormone levels may contribute to this association. A recent animal study demonstrated sex-specific differences in fat distribution and hepatic lipid accumulation in response to a fast-food diet, with the observed effects found to be more pronounced in male than in female mice [43]. Furthermore, fast-food diet-induced elevated ALT and AST levels were more prominent in male mice [43]. In human epidemiological studies, the association between dietary quality and hepatic steatosis is more pronounced among males [44]. Estrogen may play a protective role by preventing ectopic fat accumulation and reducing systemic inflammation induced by poor dietary intake [43,45,46,47]. Sex differences in dietary patterns may partially explain the pronounced association observed between diet quality and liver enzyme levels among male participants. For example, men tended to have poorer overall diet quality than women. As a result, men with higher dietary quality may experience greater protective effects against elevated liver enzyme levels. However, further research is needed to clarify the role of sex-specific dietary patterns in liver health.

Nevertheless, this study has some limitations. First, owing to its cross-sectional design, causal relationships between dietary quality and liver enzyme levels could not be established. For instance, reverse causation may be a concern; individuals with impaired liver function may be more motivated to improve their dietary quality to maintain their health, which could attenuate or obscure the true direction of the association. Longitudinal studies are required to confirm these findings. Second, our study did not account for several important factors, including exposure to hepatotoxins, environmental exposure, use of hepatotoxic medications, or genetic risk factors associated with liver function [14,48,49]. Particularly because the KNHANES is a nationwide health screening survey based on a large-scale sample, it does not include in-depth genetic testing. Therefore, genetic predisposition could not be considered in our analyses. Future studies should incorporate genetic information to better elucidate the underlying mechanisms. Third, owing to the lack of available information, we were unable to examine the association between dietary quality and a broader range of liver enzymes, such as gamma-glutamyl transferase. Future studies should explore these relationships to elicit a more comprehensive understanding of the effects of diet on liver function. Fourth, although the KHEI is an evidence-based and validated tool for assessing dietary quality among Korean adults, the dietary data used in this study were based on a single 24-hour recall. Therefore, the measurements used may not accurately reflect long-term dietary patterns and may be susceptible to recall bias. Accordingly, to improve the accuracy of future research, repeated dietary assessments and the use of dietary records should be considered. Fifth, the impact of physical exercise has not been fully validated or addressed in this study.

Despite these limitations, the use of a nationally representative sample enhances the generalizability of our findings. Moreover, the exploration of sex-based differences in the association between dietary quality and liver enzyme levels adds novelty to the literature and provides a more nuanced understanding that may guide both clinical practice and public health policy development. For instance, policies are needed to address various social and occupational factors that may negatively impact dietary quality [50,51].

## 5. Conclusions

This study demonstrated the sex differences in the association between dietary quality and elevated AST or ALT levels, with a pronounced inverse association being observed among male participants. Our study suggests the importance of dietary quality in the prevention of liver diseases, and further research is warranted to elucidate the underlying mechanisms of the observed sex-specific associations and to inform the development of targeted dietary interventions for liver health.

## Figures and Tables

**Table 1:** Sociodemographic characteristics of participants.

	Overall	Males	Females	*p* Value ^a^
	*n* = 38,297	*n* = 15,997	*n* = 22,300	
Korean Healthy Eating Index				
Mean (SD)	63.0 (13.3)	61.6 (13.0)	64.1 (13.5)	<0.001
Range (min.—max.)	13.5–99.8	15.0–99.1	13.5–99.8	
Total adequacy score				
Mean (SD)	31.5 (10.5)	30.8 (10.3)	31.9 (10.7)	<0.001
Total moderation score				
Mean (SD)	22.8 (6.0)	21.8 (6.0)	23.5 (5.8)	<0.001
Total balance score				
Mean (SD)	8.8 (4.7)	9.0 (4.7)	8.7 (4.7)	<0.001
Age (years)				
Mean (SD)	51.4 (16.7)	51.5 (17.1)	51.4 (16.4)	0.130
Range (min.—max.)	19–80	19–80	19–80	
Income level				
Lowest	7126 (18.6%)	2785 (17.4%)	4341 (19.5%)	<0.001
Low	9499 (24.8%)	3907 (24.4%)	5592 (25.1%)	
High	10,469 (27.3%)	4458 (27.9%)	6011 (27.0%)	
Highest	11,203 (29.3%)	4847 (30.3%)	6356 (28.5%)	
Educational level				
Middle school or below	11,728 (30.6%)	4048 (25.3%)	7680 (34.4%)	<0.001
High school	12,693 (33.1%)	5670 (35.4%)	7023 (31.5%)	
College or above	13,876 (36.2%)	6279 (39.3%)	7597 (34.1%)	
Marital status				
Married	31,957 (83.4%)	12,685 (79.3%)	19,272 (86.4%)	<0.001
Unmarred or others	6340 (16.6%)	3312 (20.7%)	3028 (13.6%)	
Employment status				
Workers	22,881 (59.7%)	11,377 (71.1%)	11,504 (51.6%)	<0.001
Unemployed	15,416 (40.3%)	4620 (28.9%)	10,796 (48.4%)	
Smoking status				
No	32,902 (85.9%)	11,375 (71.1%)	21,527 (96.5%)	<0.001
Yes	5395 (14.1%)	4622 (28.9%)	773 (3.5%)	
Physical activity				
No	21,422 (55.9%)	8303 (51.9%)	13,119 (58.8%)	<0.001
Yes	16,875 (44.1%)	7694 (48.1%)	9181 (41.2%)	
Excess alcohol use				
No	33,934 (88.6%)	12,864 (80.4%)	21,070 (94.5%)	<0.001
Yes	4363 (11.4%)	3133 (19.6%)	1230 (5.5%)	
Body mass index				
Mean (SD)	23.9 (3.5)	24.5 (3.4)	23.5 (3.6)	<0.001
Range (min.—max.)	13.3–62.6	15.0–50.9	13.3–62.6	
Hypertension ^b^				
No	29,768 (77.7%)	12,273 (76.7%)	17,495 (78.5%)	<0.001
Yes	8529 (22.3%)	3724 (23.3%)	4805 (21.5%)	
Diabetes ^c^				
No	34,870 (91.1%)	14,364 (89.8%)	20,506 (92.0%)	<0.001
Yes	3427 (8.9%)	1633 (10.2%)	1794 (8.0%)	
Elevated AST level				
No	36,415 (95.1%)	14,866 (92.9%)	21,549 (96.6%)	<0.001
Yes	1882 (4.9%)	1131 (7.1%)	751 (3.4%)	
Range of AST (min.—max.)	5–927	9–927	5–404	
Elevated ALT level				
No	33,678 (87.9%)	12,871 (80.5%)	20,807 (93.3%)	<0.001
Yes	4619 (12.1%)	3126 (19.5%)	1493 (6.7%)	
Range of ALT (min.—max.)	1–458	1–402	3–458	

SD, standard deviation. ^a^ Chi-squared test for categorical variables and *t*-test for continuous variables. ^b^ Current use of antihypertensive drugs. ^c^ Current use of antidiabetic drugs or insulin.

**Table 2:** Distribution of subcomponents of the Korean Healthy Eating Index and serum AST and SLT levels according to the categorization of the total Korean Healthy Eating Index score.

	Overall	KHEI Categories				
		Lowest	Low	Average	High	Highest
**Male**						
**Total KHEI score**	61.6 ± 13.0	43.6 ± 6.2	55.9 ± 2.5	63.6 ± 2.0	70.6 ± 2.2	80.5 ± 4.6
**Total adequacy score ^a^**	30.8 ± 10.3	19.7 ± 7.6	26.5 ± 6.7	31.6 ± 6.1	36.7 ± 5.5	43.6 ± 5.4
Breakfast (0–10)	7.5 ± 3.8	4.4 ± 4.2	6.9 ± 4.0	8.3 ± 3.1	9.1 ± 2.4	9.5 ± 1.6
Whole grains (0–5)	2.1 ± 2.2	0.9 ± 1.7	1.7 ± 2.1	2.2 ± 2.2	2.8 ± 2.2	3.4 ± 2.0
Total fruit (0–5)	1.9 ± 2.1	0.6 ± 1.4	1.2 ± 1.8	1.8 ± 2.0	2.6 ± 2.1	3.6 ± 1.7
Fruit, excluding juice (0–5)	2.1 ± 2.3	0.7 ± 1.6	1.3 ± 2.0	2.0 ± 2.3	2.9 ± 2.3	4.0 ± 1.9
Total vegetables (0–5)	3.8 ± 1.4	3.1 ± 1.6	3.6 ± 1.4	3.9 ± 1.3	4.2 ± 1.1	4.4 ± 1.0
Vegetable, excluding kimchi and pickles (0–5)	3.4 ± 1.6	2.5 ± 1.7	3.1 ± 1.6	3.5 ± 1.5	3.8 ± 1.4	4.2 ± 1.2
Meat, fish, eggs, and beans (0–10)	7.3 ± 3.0	6.1 ± 3.6	6.7 ± 3.2	7.3 ± 2.8	8.1 ± 2.4	8.8 ± 1.9
Milk and dairy (0–10)	2.9 ± 4.3	1.5 ± 3.3	2.1 ± 3.8	2.6 ± 4.1	3.2 ± 4.4	5.8 ± 4.6
**Total moderation score ^b^**	21.8 ± 6.0	18.1 ± 6.8	21.5 ± 6.1	22.6 ± 5.2	23.2 ± 4.8	24.6 ± 3.9
Saturated fatty acid (0–10)	7.6 ± 3.8	5.0 ± 4.6	7.4 ± 4.0	8.3 ± 3.3	8.8 ± 2.7	9.3 ± 1.9
Sodium (0–10)	5.7 ± 3.5	5.7 ± 3.7	5.7 ± 3.6	5.5 ± 3.5	5.5 ± 3.3	6.1 ± 3.0
Sweets (0–10)	8.5 ± 3.0	7.4 ± 3.8	8.5 ± 3.0	8.8 ± 2.6	8.9 ± 2.5	9.2 ± 2.1
**Total balance score ^c^**	9.0 ± 4.7	5.7 ± 4.7	7.9 ± 4.5	9.4 ± 4.2	10.7 ± 3.8	12.3 ± 3.0
Carbohydrate (0–5)	2.5 ± 2.1	1.5 ± 2.0	2.1 ± 2.1	2.6 ± 2.1	3.1 ± 2.0	3.7 ± 1.6
Fat (0–5)	3.3 ± 2.1	2.1 ± 2.2	3.0 ± 2.2	3.5 ± 2.0	3.9 ± 1.8	4.5 ± 1.2
Total energy (0–5)	3.1 ± 2.2	2.1 ± 2.3	2.8 ± 2.3	3.3 ± 2.1	3.7 ± 2.0	4.1 ± 1.7
**AST level**						
Mean (SD)	25.3 ± 16.3	26.0 ± 23.1	25.8 ± 19.7	24.9 ± 11.1	25.0 ± 10.5	24.5 ± 10.0
**ALT level**						
Mean (SD)	26.9 ± 20.0	28.9 ± 24.0	27.7 ± 21.8	25.8 ± 17.3	26.2 ± 17.4	25.2 ± 16.9
**Body Mass Index**						
Mean (SD)	24.5 ± 3.4	24.7 ± 3.8	24.5 ± 3.5	24.4 ± 3.2	24.4 ± 3.1	24.3 ± 3.1
**Female**						
**Total KHEI score**	64.1 ± 13.5	43.6 ± 6.4	56.0 ± 2.4	63.5 ± 2.0	70.8 ± 2.2	81.3 ± 5.0
**Total adequacy score ^a^**	31.9 ± 10.7	18.2 ± 7.2	26.3 ± 6.6	31.3 ± 6.1	36.6 ± 5.6	43.7 ± 5.5
Breakfast (0–10)	7.5 ± 3.7	4.1 ± 4.1	6.6 ± 4.0	7.8 ± 3.5	8.8 ± 2.6	9.5 ± 1.7
Whole grains (0–5)	2.1 ± 2.2	0.9 ± 1.6	1.6 ± 2.0	2.1 ± 2.1	2.5 ± 2.1	3.3 ± 2.0
Total fruit (0–5)	2.6 ± 2.2	1.0 ± 1.7	1.8 ± 2.1	2.6 ± 2.2	3.3 ± 2.0	4.1 ± 1.5
Fruit, excluding juice (0–5)	2.8 ± 2.4	1.1 ± 1.9	2.0 ± 2.3	2.8 ± 2.3	3.5 ± 2.2	4.3 ± 1.6
Total vegetables (0–5)	3.3 ± 1.5	2.2 ± 1.5	3.0 ± 1.5	3.4 ± 1.4	3.7 ± 1.3	4.0 ± 1.2
Vegetable, excluding kimchi and pickles (0–5)	3.2 ± 1.7	2.0 ± 1.6	2.7 ± 1.6	3.2 ± 1.6	3.6 ± 1.5	4.0 ± 1.3
Meat, fish, eggs, and beans (0–10)	6.8 ± 3.2	5.0 ± 3.5	6.0 ± 3.3	6.7 ± 3.0	7.5 ± 2.7	8.5 ± 2.2
Milk and dairy (0–10)	3.5 ± 4.4	2.0 ± 3.7	2.5 ± 4.0	2.9 ± 4.2	3.6 ± 4.4	5.9 ± 4.5
**Total moderation score ^b^**	23.5 ± 5.8	20.0 ± 6.7	22.5 ± 6.2	23.8 ± 5.6	24.4 ± 5.0	25.8 ± 4.0
Saturated fatty acid (0–10)	7.7 ± 3.8	5.4 ± 4.7	7.0 ± 4.2	8.0 ± 3.6	8.4 ± 3.1	9.0 ± 2.3
Sodium (0–10)	7.6 ± 2.9	7.9 ± 3.0	7.6 ± 3.0	7.5 ± 3.0	7.4 ± 3.0	7.7 ± 2.6
Sweets (0–10)	8.2 ± 3.3	6.7 ± 4.1	7.9 ± 3.5	8.3 ± 3.1	8.6 ± 2.9	9.0 ± 2.4
**Total balance score ^c^**	8.7 ± 4.7	5.3 ± 4.4	7.2 ± 4.6	8.4 ± 4.3	9.9 ± 4.1	11.8 ± 3.3
Carbohydrate (0–5)	2.4 ± 2.1	1.5 ± 2.0	1.9 ± 2.1	2.2 ± 2.1	2.7 ± 2.1	3.4 ± 1.8
Fat (0–5)	3.2 ± 2.1	2.1 ± 2.2	2.7 ± 2.2	3.1 ± 2.2	3.6 ± 2.0	4.3 ± 1.4
Total energy (0–5)	3.1 ± 2.2	1.8 ± 2.2	2.5 ± 2.3	3.1 ± 2.2	3.6 ± 2.0	4.1 ± 1.7
**AST level**						
Mean (SD)	21.7 ± 10.8	20.6 ± 12.4	21.5 ± 12.2	21.9 ± 9.9	21.8 ± 10.4	22.3 ± 9.4
**ALT level**						
Mean (SD)	18.5 ± 14.2	17.3 ± 15.4	18.0 ± 14.8	18.7 ± 13.5	18.7 ± 13.5	19.3 ± 13.9
**Body Mass Index**						
Mean (SD)	23.5 ± 3.6	23.3 ± 3.9	23.5 ± 3.8	23.6 ± 3.6	23.6 ± 3.5	23.4 ± 3.2

Values are presented as weighted-adjusted means ± standard deviation. ^a^ Adequacy score: Higher scores indicate greater consumption of recommended food groups such as fruits, vegetables, whole grains, protein foods, and dairy products, reflecting nutritional sufficiency. ^b^ Moderation score: Higher scores indicate limited intake of food components that should be consumed in moderation, such as saturated fats, sodium, and sweets. ^c^ Balance score: Higher scores indicate an appropriate distribution of energy intake from macronutrients, specifically carbohydrates, fat, and total energy.

**Table 3:** Association between the Korean Healthy Eating Index (KHEI) and elevated serum aspartate aminotransferase (AST) and alanine aminotransferase (ALT) levels in males and females.

	Elevated AST Level		Elevated ALT Level	
	Males	Females	Males	Females
	OR (95% CI)	OR (95% CI)	OR (95% CI)	OR (95% CI)
**KHEI (categorical)**				
Lowest	Reference	Reference	Reference	Reference
Low	0.98 (0.80–1.20)	1.09 (0.80–1.48)	0.98 (0.85–1.13)	0.91 (0.73–1.13)
Average	0.76 (0.60–0.96)	1.10 (0.82–1.46)	0.91 (0.79–1.06)	0.96 (0.78–1.18)
High	0.81 (0.64–1.01)	0.79 (0.58–1.08)	0.97 (0.83–1.12)	0.95 (0.78–1.17)
Highest	0.67 (0.52–0.88)	1.05 (0.78–1.41)	0.80 (0.68–0.96)	1.01 (0.83–1.24)
**KHEI (continuous)**				
10-point increase	0.90 (0.85–0.96)	0.98 (0.91–1.05)	0.96 (0.92–1.00)	1.00 (0.95–1.05)

OR, odds ratio; CI, confidence interval. All models were adjusted for age, income, education, marital status, employment status, smoking, physical activity, alcohol use, body mass index, hypertension, and diabetes.

## Data Availability

Data are available at https://knhanes.kdca.go.kr/knhanes (access date: 1 May 2025).

## Supplementary Materials

The following supporting information can be downloaded at https://www.mdpi.com/article/10.3390/nu17142372/s1: Table S1. Distribution of subcomponents of the Korean Healthy Eating Index according to sex; Table S2. Association between the Korean Healthy Eating Index (KHEI) and elevated serum aspartate aminotransferase (AST) and alanine aminotransferase (ALT) levels among the overall sample; Table S3. Association between the Korean Healthy Eating Index (KHEI) and elevated serum aspartate aminotransferase (AST) and alanine aminotransferase (ALT) levels in males and females. Sensitivity analyses using imputed datasets.
