# Supplementary material for: Sex Differences in the Association Between the Korean Healthy Eating Index and Liver Enzymes Among Korean Adults
Source: Nutrients. 2025 Jul 20;17(14):2372. doi: 10.3390/nu17142372 (PMC12298670; doi:10.3390/nu17142372)
Supplement: Supplementary file 1 [file nutrients-17-02372-s001.zip › nutrients-3742935-supplementary.pdf]

Table S1. Distribution of subcomponents of the Korean Healthy Eating Index according to sex

	**Male**	**Female**
**Total KHEI score**	61.6±13.0	64.1±13.5
**Total adequacy score**	30.8±10.3	31.9±10.7
Breakfast (0–10)	7.5±3.8	7.5±3.7
Whole grains (0–5)	2.1±2.2	2.1±2.2
Total fruit (0–5)	1.9±2.1	2.6±2.2
Fruit, excluding juice (0–5)	2.1±2.3	2.8±2.4
Total vegetables (0–5)	3.8±1.4	3.3±1.5
Vegetable, excluding kimchi and pickles (0–5)	3.4±1.6	3.2±1.7
Meat, fish, eggs, and beans (0–10)	7.3±3.0	6.8±3.2
Milk and dairy (0–10)	2.9±4.3	3.5±4.4
**Total moderation score**	21.8±6.0	23.5±5.8
Saturated fatty acid (0–10)	7.6±3.8	7.7±3.8
Sodium (0–10)	5.7±3.5	7.6±2.9
Sweets (0–10)	8.5±3.0	8.2±3.3
**Total balance score**	9.0±4.7	8.7±4.7
Carbohydrate (0–5)	2.5±2.1	2.4±2.1
Fat (0–5)	3.3±2.1	3.2±2.1
Total energy (0–5)	3.1±2.2	3.1±2.2

Table S2 Association between the Korean Healthy Eating Index (KHEI) and elevated serum aspartate aminotransferase (AST) and alanine aminotransferase (ALT) levels among the overall sample

	Elevated AST level		Elevated ALT level	
	**Model 1**	**Model 2**	**Model 1**	**Model 2**
	**OR (95% CI)**	**OR (95% CI)**	**OR (95% CI)**	**OR (95% CI)**
KHEI				
10-point increase	0.93 (0.89–0.97)	0.89 (0.84–0.94)	0.97 (0.94–1.00)	0.94 (0.90–0.97)
Sex				
Male	Reference	Reference	Reference	Reference
Female	0.49 (0.43–0.55)	0.21 (0.12–0.36)	0.30 (0.27–0.33)	0.13 (0.09–0.19)
Sex × KHEI				
Female × KHEI		1.15 (1.06–1.25)[a]		1.15 (1.08–1.22)[b]

OR, odds ratio; CI, confidence interval;

Model 1, fully adjusted model.

Model 2, Model 1 + interaction term (sex × KHEI).

[a] $p = 0.001$

[b] $p < 0.001$

Table S3 Association between the Korean Healthy Eating Index (KHEI) and elevated serum aspartate aminotransferase (AST) and alanine aminotransferase (ALT) levels in males and females. Sensitivity analyses using imputed datasets.

	**Elevated AST level**		**Elevated ALT level**	
	**Males**	**Females**	**Males**	**Females**
	**OR (95% CI)**	**OR (95% CI)**	**OR (95% CI)**	**OR (95% CI)**
**KHEI (categorical)**				
Lowest	Reference	Reference	Reference	Reference
Low	0.98 (0.81–1.19)	0.99 (0.74–1.33)	1.01 (0.88–1.16)	0.89 (0.72–1.09)
Average	0.81 (0.65–1.00)	0.98 (0.74–1.28)	0.93 (0.81–1.07)	0.95 (0.77–1.15)
High	0.81 (0.66–1.01)	0.77 (0.57–1.03)	0.98 (0.85–1.13)	0.94 (0.78–1.15)
Highest	0.71 (0.56–0.91)	1.00 (0.75–1.31)	0.83 (0.70–0.98)	1.02 (0.84–1.24)

OR, odds ratio; CI, confidence interval.

All models were adjusted for age, income, education, marital status, employment status, smoking, physical activity, alcohol use, body mass index, hypertension, and diabetes.
